# *N*-(4-Chloro-2-nitro­phen­yl)-*N*-(methyl­sulfon­yl)acetamide

**DOI:** 10.1107/S1600536808032157

**Published:** 2008-10-11

**Authors:** Muhammad Zia-ur-Rehman, Nosheen Akbar, Muhammad Nadeem Arshad, Islam Ullah Khan

**Affiliations:** a Applied Chemistry Research Centre, PCSIR Laboratories Complex, Lahore 54600, Pakistan; b Centre for High Energy Physics, University of the Punjab, Lahore 54590, Pakistan; c Department of Chemistry, Government College University, Lahore 54000, Pakistan

## Abstract

The title compound, C_9_H_9_ClN_2_O_5_S, is of inter­est as a precursor to biologically active substituted quinolines and related compounds. The structure displays inter­molecular C—H⋯O inter­actions. Each mol­ecule is linked to two adjacent neighbours *via* weak centrosymmetric dimer-forming inter­actions, forming chains in the [101] direction.

## Related literature

For synthesis and biological evaluation of sulfur-containing heterocyclic compounds, see: Zia-ur-Rehman *et al.* (2005 ▶, 2006 ▶, 2007 ▶, 2008 ▶); Wen *et al.* (2005 ▶); Zhang, Xu, Wen *et al.* (2006 ▶). For related mol­ecules, see: (Wen *et al.*, 2006 ▶; Zhang, Xu, Zou *et al.* (2006 ▶). For bond-length data, see: Allen *et al.* (1987 ▶).
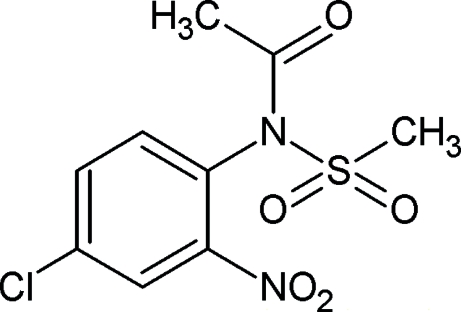


## Experimental

### 

#### Crystal data

C_9_H_9_ClN_2_O_5_S
*M* *_r_* = 292.70
Monoclinic, 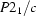

*a* = 9.8071 (4) Å
*b* = 9.4310 (4) Å
*c* = 13.5679 (7) Å
β = 105.883 (2)°
*V* = 1207.00 (9) Å^3^
*Z* = 4
Mo *K*α radiation
μ = 0.50 mm^−1^
*T* = 296 (2) K
0.25 × 0.15 × 0.09 mm

#### Data collection

Bruker APEXII CCD area-detector diffractometer
Absorption correction: multi-scan (*SADABS*; Sheldrick, 1996 ▶) *T*_min_ = 0.913, *T*_max_ = 0.956
13383 measured reflections
2988 independent reflections
2077 reflections with *I* > 2σ(*I*)
*R*_int_ = 0.043

#### Refinement

*R*[*F* ^2^ > 2σ(*F* ^2^)] = 0.040
*wR*(*F* ^2^) = 0.104
*S* = 1.02
2981 reflections
163 parameters
H-atom parameters constrained
Δρ_max_ = 0.43 e Å^−3^
Δρ_min_ = −0.31 e Å^−3^

### 

Data collection: *SMART* (Bruker, 2007 ▶); cell refinement: *SAINT* (Bruker, 2007 ▶); data reduction: *SAINT*; program(s) used to solve structure: *SHELXS97* (Sheldrick, 2008 ▶); program(s) used to refine structure: *SHELXL97* (Sheldrick, 2008 ▶); molecular graphics: *SHELXTL* (Sheldrick, 2008 ▶); software used to prepare material for publication: *WinGX* (Farrugia, 1999 ▶) and *PLATON* (Spek, 2003 ▶).

## Supplementary Material

Crystal structure: contains datablocks I, global. DOI: 10.1107/S1600536808032157/ez2142sup1.cif

Structure factors: contains datablocks I. DOI: 10.1107/S1600536808032157/ez2142Isup2.hkl

Additional supplementary materials:  crystallographic information; 3D view; checkCIF report

## Figures and Tables

**Table 1:** Hydrogen-bond geometry (Å, °)

*D*—H⋯*A*	*D*—H	H⋯*A*	*D*⋯*A*	*D*—H⋯*A*
C2—H2⋯O5^i^	0.93	2.55	3.404 (3)	153
C9—H9*B*⋯O3^ii^	0.96	2.58	3.521 (3)	169

Symmetry codes: (i) 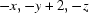; (ii) 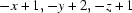.

**Fig. 1.**
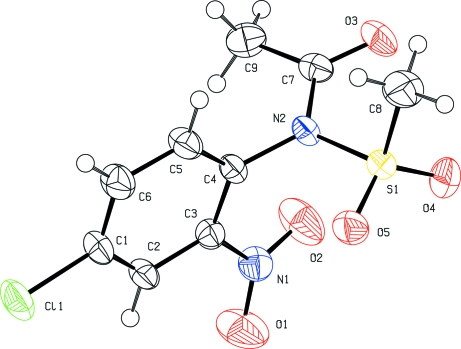
The asymmetric unit of the title compound showing the atom labelling scheme. Displacement ellipsoids are drawn at the 50% probability level.

**Fig. 2.**
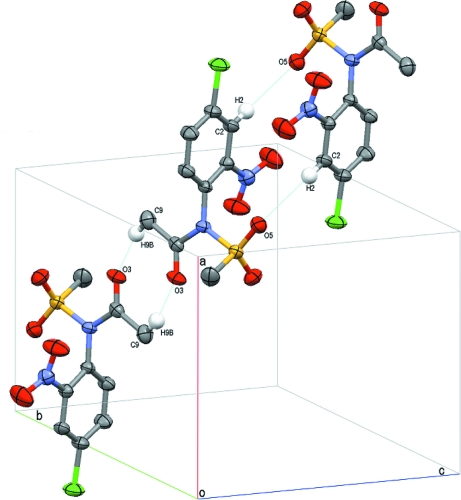
Perspective view of a portion of the crystal packing, viewed approximately down the b-axis, showing hydrogen bond interactions (dashed lines) along the [101] direction. H atoms not involved in hydrogen bonding have been omitted for clarity.

## supplementary crystallographic information

### Comment

*N*-(Substituted phenyl)acetamides are well known for their importance as intermediates in organic synthesis. They are used as precursors for the synthesis of many heterocyclic compounds, *e.g.* 2,5-piperazinedione (Wen *et al.*, 2006), (quinolin-8-yloxy)acetamide (Zhang, Xu, Wen *et al.*, 2006) and 2,2-(1,3,4-thiadiazolyl-2,5-dithio)diacetamide (Wen *et al.*, 2005). In the present paper, the structure of *N*-(4-chloro-2-nitrophenyl)-*N*-(methylsulfonyl)acetamide (I) has been determined as part of a research program involving the synthesis and biological evaluation of sulfur containing heterocyclic compounds (Zia-ur-Rehman *et al.*, 2005, 2006, 2007, 2008).

In the molecule of I (Fig. 1), the bond lengths and bond angles are similar to those in related molecules (Wen *et al.*, 2006; Zhang, Xu, Zou *et al.*, 2006) and are within normal ranges (Allen *et al.*, 1987). The nitro group is slightly twisted out of the plane of the benzene ring, as indicated by O1—N1—C3—C2 and O2—N1—C3—C2 torsion angles of -16.7 (3) and 160.9 (2)°, respectively. Each molecule is linked to its neighbour *via* a centrosymmetric head-to-tail interaction between the methyl hydrogen H9B and the carbonyl oxygen [C9—H9B···O3]. Adjacent pairs of these molecules are then linked into chains *via* intermolecular [C2—H5···O5] interactions along the [101] direction (Table 1 and Fig. 2).

### Experimental

A mixture of *N*-(4-chloro-2-nitrophenyl)methane sulfonamide (2.507 g; 10.0 mmoles) and acetic anhydride (10.0 ml) was heated to reflux for half an hour and then poured over crushed ice. Resultant solids were then washed with cold water and dried under reduced pressure. Yellow crystals were obtained by slow evaporation of an ethanolic solution over a period of two days.

### Refinement

H atoms bound to C were placed in geometric positions (C—H distance = 0.95 Å) using a riding model with *U*_iso_(H) = 1.2 *U*_eq_(C).

### Crystal data

**Table:**

C_9_H_9_ClN_2_O_5_S	*F*(000) = 600
*M**_r_* = 292.70	*D*_x_ = 1.611 Mg m^−^^3^
Monoclinic, *P*2_1_/*c*	Melting point: 401 K
Hall symbol: -P 2ybc	Mo *K*α radiation, λ = 0.71073 Å
*a* = 9.8071 (4) Å	Cell parameters from 3121 reflections
*b* = 9.4310 (4) Å	θ = 2.7–27.2°
*c* = 13.5679 (7) Å	µ = 0.50 mm^−^^1^
β = 105.883 (2)°	*T* = 296 K
*V* = 1207.00 (9) Å^3^	Needle, light yellow
*Z* = 4	0.25 × 0.15 × 0.09 mm

### Data collection

**Table:**

Bruker APEXII CCD area-detector diffractometer	2988 independent reflections
Radiation source: fine-focus sealed tube	2077 reflections with *I* > 2σ(*I*)
graphite	*R*_int_ = 0.043
Detector resolution: 7.5 pixels mm^-1^	θ_max_ = 28.3°, θ_min_ = 2.7°
φ and ω scans	*h* = −12→13
Absorption correction: multi-scan (SADABS; Sheldrick, 1996)	*k* = −12→12
*T*_min_ = 0.913, *T*_max_ = 0.956	*l* = −18→17
13383 measured reflections	

### Refinement

**Table:**

Refinement on *F*^2^	Primary atom site location: structure-invariant direct methods
Least-squares matrix: full	Secondary atom site location: difference Fourier map
*R*[*F*^2^ > 2σ(*F*^2^)] = 0.040	Hydrogen site location: inferred from neighbouring sites
*wR*(*F*^2^) = 0.104	H-atom parameters constrained
*S* = 1.02	*w* = 1/[σ^2^(*F*_o_^2^) + (0.0426*P*)^2^ + 0.5587*P*] where *P* = (*F*_o_^2^ + 2*F*_c_^2^)/3
2981 reflections	(Δ/σ)_max_ < 0.001
163 parameters	Δρ_max_ = 0.43 e Å^−^^3^
0 restraints	Δρ_min_ = −0.31 e Å^−^^3^

### Special details

**Table:**

Geometry. All e.s.d.'s (except the e.s.d. in the dihedral angle between two l.s. planes) are estimated using the full covariance matrix. The cell e.s.d.'s are taken into account individually in the estimation of e.s.d.'s in distances, angles and torsion angles; correlations between e.s.d.'s in cell parameters are only used when they are defined by crystal symmetry. An approximate (isotropic) treatment of cell e.s.d.'s is used for estimating e.s.d.'s involving l.s. planes.
Refinement. Refinement of *F*^2^ against ALL reflections. The weighted *R*-factor *wR* and goodness of fit *S* are based on *F*^2^, conventional *R*-factors *R* are based on *F*, with *F* set to zero for negative *F*^2^. The threshold expression of *F*^2^ > σ(*F*^2^) is used only for calculating *R*-factors(gt) *etc*. and is not relevant to the choice of reflections for refinement. *R*-factors based on *F*^2^ are statistically about twice as large as those based on *F*, and *R*- factors based on ALL data will be even larger.

### Fractional atomic coordinates and isotropic or equivalent isotropic displacement parameters (Å^2^)

**Table:**

	*x*	*y*	*z*	*U*_iso_*/*U*_eq_	
S1	0.43935 (5)	0.98058 (6)	0.14520 (4)	0.03097 (15)	
Cl1	−0.20712 (6)	0.67766 (7)	−0.00125 (6)	0.0511 (2)	
N2	0.34896 (17)	0.91810 (19)	0.22582 (14)	0.0276 (4)	
O5	0.34507 (18)	0.96150 (18)	0.04574 (13)	0.0439 (4)	
C4	0.2107 (2)	0.8605 (2)	0.17969 (16)	0.0269 (5)	
O3	0.52800 (17)	0.97095 (19)	0.36537 (13)	0.0454 (4)	
N1	0.0872 (2)	1.0934 (2)	0.18329 (16)	0.0361 (5)	
C3	0.0874 (2)	0.9425 (2)	0.15362 (17)	0.0283 (5)	
O1	−0.0130 (2)	1.1651 (2)	0.13957 (18)	0.0695 (7)	
C1	−0.0476 (2)	0.7464 (3)	0.07164 (19)	0.0365 (5)	
O4	0.49004 (18)	1.11878 (17)	0.17659 (15)	0.0477 (5)	
O2	0.1842 (2)	1.1381 (2)	0.25205 (18)	0.0651 (6)	
C5	0.1996 (2)	0.7188 (2)	0.15196 (19)	0.0359 (5)	
H5	0.2800	0.6618	0.1689	0.043*	
C6	0.0708 (2)	0.6607 (3)	0.09955 (19)	0.0401 (6)	
H6	0.0639	0.5647	0.0833	0.048*	
C7	0.4104 (2)	0.9221 (2)	0.33238 (17)	0.0315 (5)	
C2	−0.0409 (2)	0.8874 (2)	0.09933 (18)	0.0327 (5)	
H2	−0.1215	0.9441	0.0816	0.039*	
C8	0.5815 (3)	0.8637 (3)	0.1615 (2)	0.0501 (7)	
H8A	0.5463	0.7690	0.1460	0.075*	
H8B	0.6390	0.8679	0.2312	0.075*	
H8C	0.6374	0.8899	0.1163	0.075*	
C9	0.3259 (3)	0.8606 (3)	0.39765 (19)	0.0415 (6)	
H9A	0.2285	0.8887	0.3716	0.062*	
H9B	0.3621	0.8944	0.4666	0.062*	
H9C	0.3323	0.7591	0.3969	0.062*	

### Atomic displacement parameters (Å^2^)

**Table:**

	*U*^11^	*U*^22^	*U*^33^	*U*^12^	*U*^13^	*U*^23^
S1	0.0248 (3)	0.0320 (3)	0.0347 (3)	0.0000 (2)	0.0057 (2)	0.0040 (2)
Cl1	0.0311 (3)	0.0557 (4)	0.0567 (4)	−0.0129 (3)	−0.0043 (3)	−0.0082 (3)
N2	0.0209 (8)	0.0327 (9)	0.0269 (10)	−0.0027 (7)	0.0024 (7)	−0.0027 (8)
O5	0.0386 (9)	0.0575 (11)	0.0322 (10)	−0.0048 (8)	0.0039 (8)	0.0063 (8)
C4	0.0208 (9)	0.0328 (11)	0.0253 (11)	−0.0021 (8)	0.0032 (8)	−0.0017 (9)
O3	0.0317 (9)	0.0564 (11)	0.0388 (10)	−0.0073 (8)	−0.0064 (8)	−0.0053 (8)
N1	0.0334 (10)	0.0337 (10)	0.0424 (12)	0.0039 (8)	0.0123 (9)	−0.0019 (9)
C3	0.0268 (10)	0.0283 (11)	0.0292 (12)	−0.0009 (8)	0.0066 (9)	−0.0012 (9)
O1	0.0571 (13)	0.0447 (11)	0.0897 (17)	0.0213 (10)	−0.0088 (12)	−0.0094 (11)
C1	0.0256 (11)	0.0426 (13)	0.0364 (14)	−0.0069 (10)	0.0002 (10)	−0.0021 (10)
O4	0.0457 (10)	0.0354 (9)	0.0607 (13)	−0.0111 (8)	0.0124 (9)	0.0025 (8)
O2	0.0447 (11)	0.0471 (11)	0.0889 (17)	0.0022 (9)	−0.0063 (11)	−0.0287 (11)
C5	0.0277 (11)	0.0326 (11)	0.0422 (15)	0.0037 (9)	0.0010 (10)	−0.0035 (10)
C6	0.0368 (13)	0.0329 (12)	0.0447 (15)	−0.0034 (10)	0.0012 (11)	−0.0067 (11)
C7	0.0308 (11)	0.0307 (11)	0.0291 (13)	0.0039 (9)	0.0016 (10)	−0.0025 (9)
C2	0.0202 (10)	0.0403 (12)	0.0356 (13)	0.0008 (9)	0.0043 (9)	0.0024 (10)
C8	0.0370 (14)	0.0579 (17)	0.0606 (19)	0.0155 (12)	0.0219 (13)	0.0118 (14)
C9	0.0489 (14)	0.0445 (14)	0.0309 (14)	0.0029 (11)	0.0104 (12)	0.0004 (11)

### Geometric parameters (Å, °)

**Table:**

S1—O4	1.4182 (17)	C1—C2	1.379 (3)
S1—O5	1.4232 (18)	C1—C6	1.380 (3)
S1—N2	1.6913 (19)	C5—C6	1.382 (3)
S1—C8	1.743 (2)	C5—H5	0.9300
Cl1—C1	1.732 (2)	C6—H6	0.9300
N2—C7	1.406 (3)	C7—C9	1.487 (3)
N2—C4	1.435 (2)	C2—H2	0.9300
C4—C5	1.384 (3)	C8—H8A	0.9600
C4—C3	1.397 (3)	C8—H8B	0.9600
O3—C7	1.208 (3)	C8—H8C	0.9600
N1—O1	1.207 (2)	C9—H9A	0.9600
N1—O2	1.212 (3)	C9—H9B	0.9600
N1—C3	1.479 (3)	C9—H9C	0.9600
C3—C2	1.374 (3)		
			
O4—S1—O5	118.97 (11)	C4—C5—H5	119.5
O4—S1—N2	109.18 (10)	C1—C6—C5	119.4 (2)
O5—S1—N2	104.46 (9)	C1—C6—H6	120.3
O4—S1—C8	109.91 (13)	C5—C6—H6	120.3
O5—S1—C8	109.27 (13)	O3—C7—N2	119.2 (2)
N2—S1—C8	103.88 (11)	O3—C7—C9	124.0 (2)
C7—N2—C4	123.11 (18)	N2—C7—C9	116.74 (19)
C7—N2—S1	120.17 (14)	C3—C2—C1	118.6 (2)
C4—N2—S1	116.71 (14)	C3—C2—H2	120.7
C5—C4—C3	117.84 (19)	C1—C2—H2	120.7
C5—C4—N2	118.61 (18)	S1—C8—H8A	109.5
C3—C4—N2	123.35 (18)	S1—C8—H8B	109.5
O1—N1—O2	123.1 (2)	H8A—C8—H8B	109.5
O1—N1—C3	117.83 (19)	S1—C8—H8C	109.5
O2—N1—C3	119.05 (19)	H8A—C8—H8C	109.5
C2—C3—C4	121.92 (19)	H8B—C8—H8C	109.5
C2—C3—N1	116.14 (18)	C7—C9—H9A	109.5
C4—C3—N1	121.95 (18)	C7—C9—H9B	109.5
C2—C1—C6	121.1 (2)	H9A—C9—H9B	109.5
C2—C1—Cl1	119.02 (18)	C7—C9—H9C	109.5
C6—C1—Cl1	119.86 (18)	H9A—C9—H9C	109.5
C6—C5—C4	121.1 (2)	H9B—C9—H9C	109.5
C6—C5—H5	119.5		
			
O4—S1—N2—C7	−50.92 (18)	O1—N1—C3—C4	163.6 (2)
O5—S1—N2—C7	−179.19 (16)	O2—N1—C3—C4	−18.8 (3)
C8—S1—N2—C7	66.30 (19)	C3—C4—C5—C6	0.5 (4)
O4—S1—N2—C4	130.04 (16)	N2—C4—C5—C6	−174.5 (2)
O5—S1—N2—C4	1.77 (17)	C2—C1—C6—C5	−3.1 (4)
C8—S1—N2—C4	−112.74 (18)	Cl1—C1—C6—C5	175.8 (2)
C7—N2—C4—C5	−91.8 (3)	C4—C5—C6—C1	2.1 (4)
S1—N2—C4—C5	87.2 (2)	C4—N2—C7—O3	179.2 (2)
C7—N2—C4—C3	93.5 (3)	S1—N2—C7—O3	0.2 (3)
S1—N2—C4—C3	−87.5 (2)	C4—N2—C7—C9	1.0 (3)
C5—C4—C3—C2	−2.1 (3)	S1—N2—C7—C9	−178.00 (16)
N2—C4—C3—C2	172.6 (2)	C4—C3—C2—C1	1.2 (4)
C5—C4—C3—N1	177.6 (2)	N1—C3—C2—C1	−178.6 (2)
N2—C4—C3—N1	−7.7 (3)	C6—C1—C2—C3	1.5 (4)
O1—N1—C3—C2	−16.7 (3)	Cl1—C1—C2—C3	−177.41 (18)
O2—N1—C3—C2	160.9 (2)		

### Hydrogen-bond geometry (Å, °)

**Table:**

*D*—H···*A*	*D*—H	H···*A*	*D*···*A*	*D*—H···*A*
C2—H2···O5^i^	0.93	2.55	3.404 (3)	153
C9—H9B···O3^ii^	0.96	2.58	3.521 (3)	169

Symmetry codes: (i) −*x*, −*y*+2, −*z*; (ii) −*x*+1, −*y*+2, −*z*+1.
